# Excavating novel diagnostic and prognostic long non-coding RNAs (lncRNAs) for head and neck squamous cell carcinoma: an integrated bioinformatics analysis of competing endogenous RNAs (ceRNAs) and gene co-expression networks

**DOI:** 10.1080/21655979.2021.2003925

**Published:** 2021-12-21

**Authors:** Liu Yang, Pingan Lu, Xiaohui Yang, Kaiguo Li, Xuxia Chen, Song Qu

**Affiliations:** a Department of Radiation Oncology, Guangxi Medical University Cancer Hospital, Nanning, China; b Faculty of Medicine, Amsterdam UMC, Univ of Amsterdam, Amsterdam, Netherlands; c Key Laboratory of High-Incidence Tumor Prevention & Treatment, Ministry of Education, Guangxi Medical University, Nanning, China

**Keywords:** Head and neck squamous cell carcinoma, lncRNA, biomarkers, ceRNA network, WGCNA

## Abstract

Long non-coding RNAs (lncRNAs) have been demonstrated to fine-tune gene regulations that govern a broad spectrum of oncogenic processes. Nonetheless, our understanding of the roles of lncRNAs and their interactions with miRNAs and mRNAs in HNSCC is still highly rudimentary. Here, we present a comprehensive bioinformatics analysis in which competing endogenous RNA (ceRNA) network construction and weighted gene co-expression network analysis (WGCNA) were combined to explore novel diagnostic and prognostic lncRNAs for HNSCC. Differentially expressed mRNAs (DEGs), miRNAs (DEMs) and lncRNAs (DELs) were identified based on the RNA sequencing data and clinical data retrieved from TCGA database. LncRNA-regulated ceRNA networks were constructed based on the interactive RNA pairs predicted by miRDB, miRcode and TargetScan. WGCNA was conducted to identify lncRNAs that were significantly correlated with patient overall survival (OS) and HNSCC tumor. RT-qPCR was employed to validate the expression of lncRNAs in HNSCC cell lines and patient sera. A ceRNA network consisting of 90 DEGs, 7 DEMs and 67 DELs associated with clinical traits was established. WGCNA and Kaplan-Meier survival analysis revealed that 5 DELs (MIR4435-2 HG, CASC9, LINC01980, STARD4-AS1 and MIR99AHG) were significantly correlated with OS of HNSCC patients, whereas DEL PART1 was most significantly correlated with the HNSCC tumor. The *in silico* predicted expression patterns of PART1, LINC01980 and MIR4435-2 HG were further validated in HNSCC cell lines and patient sera. Collectively, the present study provided novel insights into the lncRNA-regulated ceRNA networks in HNSCC and identified novel lncRNAs that harbor diagnostic and prognostic potentials for HNSCC.

**Abbreviations** BP, biological process. CC, cellular component. ceRNA, competing endogenous RNA. DEG, differential expressions of mRNA. DEL, differentially expressed lncRNA. DEM, differentially expressed miRNA. ESCC, esophageal squamous cell carcinoma. FPKM, Fragments Per Kilobase Million. GO, Gene Ontology. GS, gene significance. HNSCC, head and neck squamous cell carcinoma. KEGG, Kyoto Encyclopedia of Genes and Genomes. LncRNA, long non-coding RNA. MCC, Maximal Clique Centrality. ME, module eigengenes. MF, molecular functions. MM, module membership. MRE, miRNA-binding site. MYO5A, Myosin-Va. PART1, prostate androgen-regulated transcript 1. RBM3, RNA‑binding motif protein 3. TCGA, The Cancer Genome Atlas. TOM, topological overlap measure. TSCC, tongue squamous cell carcinoma. WGCNA, weighted gene co-expression network analysis.

## Introduction

As the sixth most common cancer worldwide, head and neck squamous cell carcinoma (HNSCC) is a malignant neoplasm of epithelial origin that often occurs in the nasopharynx, oropharynx, laryngo-pharynx, and neck[1]. The disease is prevalent in South and Southeast Asia, accounting for an average of 550,000 new cases and more than 300,000 deaths annually [2]. HNSCC patients with early-stage disease have a favorable prognosis after curative surgical resection combined with postoperative adjuvant radiotherapy. However, due to the subclinical nature of HNSCC in its initial stages, more than 60% of the patients present with locally advanced disease at detection, which is associated with a poor 5-year overall survival rate of less than 50% [3,4]. Excavating novel biomarkers with good diagnostic potential is therefore of considerable importance for clinical outcomes of HNSCC patients. In addition, patients with advanced disease generally require multimodal treatments, and appropriate treatment decisions are critical for the survival of these patients [5,6]. Nonetheless, there is currently a lack of prognostic biomarkers with sufficient robustness and accuracy to guide tailoring current treatment modalities for individual patients [4].

Accumulating evidence has demonstrated that long non-coding RNAs (lncRNAs) play a crucial role in the pathogenesis and progression of a wide array of malignant diseases [7], which underscores the great potential of lncRNAs as a new class of cancer biomarkers and as attractive therapeutic targets for anti-cancer therapies. LncRNA is defined as an RNA transcript that is more than 200 nucleotides in length and does not overlap with the annotated coding gene [8]. Classical lncRNAs have similar structures to that of mRNAs, with 5ʹ-ended m^7^G caps and 3ʹ-ended poly(A) tails [8]. Initially, LncRNAs were regarded as ‘junk genes’. However, with the development of high-throughput sequencing technologies, many lncRNAs have been functionally characterized, participating in a broad spectrum of physiological and pathological processes such as cell cycle regulation, malignant transformation, proliferation signaling, apoptosis and invasion and metastasis of cancer cells [9–11]. Furthermore, mounting evidence has indicated that lncRNAs are capable of modulating epigenetic mechanisms, such as chromatin remodeling and histone modification [9,12,13].

A pivotal advance in understanding lncRNA biology is the competing endogenous RNA (ceRNA) theory, first postulated by Salmena et al. in 2011 [14]. These investigators proposed that lncRNAs contain miRNA-binding sites (MREs) that could function as molecular sponges to sequester miRNAs, thereby fine-tuning miRNA-regulated mRNA expressions [14] and ultimately contributing to cancer development [15,16].

Weighted gene co-expression network analysis (WGCNA) is a widely used *in silico* method that aims to identify co-expressed gene modules and to investigate the correlation between a specific gene network and the phenotype of interest [17]. WGCNA focuses on genome-wide information, constructs co-expression modules using hierarchical clustering, and can identify biologically relevant associations between modules and phenotypes of cancer [17,18].

The aim of this study is to explore novel lncRNA biomarkers with diagnostic and prognostic values for HNSCC by combining ceRNA network analysis with WGCNA, based on The Cancer Genome Atlas (TCGA) database [19]. A lncRNA-miRNA-mRNA interactive ceRNA network was constructed with the significantly differentially expressed RNAs, and the top-ranked hub RNAs of this network were subjected to the construction of a ceRNA subnetwork. Functional enrichment analyses were conducted to study the biological processes and pathways in which the differentially expressed RNAs were enriched. The diagnostic and prognostic value of the lncRNAs were analyzed with WGCNA and Kaplan-Meier survival analysis, and the expression patterns of three eligible candidate lncRNAs (PART1, MIR4435-2 HG and LINC01980) were further validated in HNSCC cell lines and patient sera. Collectively, this study identified a set of differentially expressed lncRNAs with diagnostic and prognostic values for HNSCC and constructed novel ceRNA networks in HNSCC which may shed light on the mechanism underlying the development and progression of the disease.

## Materials and methods

### Data collection and processing

The RNA sequencing data, miRNA sequencing data and the corresponding clinical data of HNSCC patients were retrieved from TCGA data repository (https://portal.gdc.cancer.gov) [20], which contained 519 HNSCC tissues and 44 adjacent normal tissues. All RNA-seq data were open data whose protocols comply with TCGA guidelines and require no further ethical approval. GDCRNATools package and DT package in R [21] (version 3.6.1) were used to read the raw data and remove repetitive samples. The data were then subjected to background correction using EdgeR package [22] in R.

### Identification and validation of differentially expressed mRNAs, miRNAs and lncRNAs

Limma package [22] was used to identify differentially expressed mRNAs (DEGs), differentially expressed miRNAs (DEMs) and differentially expressed lncRNA (DELs) between HNSCC tissues and adjacent normal tissues. A gene was considered differentially expressed when the FDR value was less than 0.01 (FDR < 0.01) and the fold change (FC) is greater than 1 (| log2 (FC) |> 1).

Gene Expression Profiling and Interactive Analyses (GEPIA, http://gepia.cancer-pku.cn) is an interactive web-based tool for analyzing RNA sequencing data of tumors and normal tissues from TCGA and genotype-tissue expression (GTEx) database [23,24]. GEPIA database was used to validate the expression of key RNAs in the constructed ceRNA networks between HNSCC and non-HNSCC normal tissues. Genes with |log_2_FC| > 1 and p-value < 0.05 were considered statistically significant.

### Construction of lncRNA-miRNA-mRNA networks

The lncRNA-miRNA-mRNA network was constructed in three steps. First, miRcode [25] (http://www.mircode.org) database and StarBase database [26] (version 2.0, http://starbase.sysu.edu.cn) were used to predict interacting DEL-DEM pairs. Next, the corresponding target genes of the predicted DEMs were retrieved from miRDB and TargetScan [27], after which the intersection was taken between the retrieved target genes and the previously identified DEGs. Finally, Cytoscape (version 3.6.1) [28] was used to visualize the ceRNA networks. The network diagram visually displayed the relationships between DELs, DEMs and DEGs, with each node representing a different RNA molecule and each edge representing the interaction between two linked nodes. The top 10 hub RNAs were selected from the constructed ceRNA network for the construction of a ceRNA subnetwork using the plugin CytoHubba [29].

### WGCNA

A weighted gene co-expression network associated with clinical traits was constructed using WGCNA package based on R [17]. Outliers were first excluded via sample clustering. A soft threshold was then applied to determine the weight of the edges between genes and to merge individual genes into gene modules. By setting 3 as the soft threshold, a weighted co-expression network was established using WGCNA package, which was a scale-free topology network. Subsequently, the gene modules were identified using the methods of Fragments Per Kilobase Million (FPKM) and topological overlap measure (TOM) [30]. To avoid generating too many modules, dynamic tree cutting algorithm [31] was used to merge modules. A minimum height of 0.6 and a minimum module size (minModuleSize) of 30 were used as cutoff values. Ultimately, the lncRNA modules that statistically significantly correlated with patient survival were selected as the modules of interest for further analysis.

### Functional enrichment analysis

Gene Ontology (GO) [25] and Kyoto Encyclopedia of Genes and Genomes (KEGG) [32] were used to study the biological processes (BP), cellular components (CC), molecular functions (MF) and pathways of the total identified DEGs and DEGs in the constructed ceRNA network. Using the R package clusterProfiler [33] and pathview [34], GO terms or KEGG pathways with an adjusted p-value < 0.05 were considered statistically significantly enriched.

### Survival analysis

Kaplan-Meier survival analysis [35] was performed to investigate the relationship between the expression of DELs and OS of HNSCC patients. The constructed ceRNA network was first combined with clinical data of HNSCC patients. The ggplot2 package [31] in R was used to generate Kaplan-Meier survival curves to evaluate the relationship between OS and the expression levels of DEGs, DEMs and DELs.

### Cell lines and blood samples

The nasopharyngeal carcinoma cell line CNE-2 was purchased from the Fudan University Shanghai Cancer Center. The nasopharyngeal carcinoma cell line C666-1, human oral keratinocytes (HOK) cell line and SCC-4 oral squamous cell carcinoma (OSCC) cell line were provided by the Experimental Center of Guangxi Medical University. The immortalized nasopharyngeal epithelial cell line NP69 was kindly provided by Professor S.W. Tsao, University of Hong Kong. The blood samples were collected from 10 patients with primary HNSCC hospitalized in Guangxi Medical University Cancer Hospital (Guangxi, China) from July 2020 to April 2021. These patients included 4 cases of oral squamous cell carcinoma, 2 cases of oropharyngeal squamous cell carcinoma, 3 cases of hypopharyngeal squamous cell carcinoma and 1 case of laryngeal squamous cell carcinoma. The blood samples of another 10 normal individuals were collected at Guangxi Medical University Cancer Hospital during the same period, which constituted the control group. All enrolled patients were diagnosed as primary HNSCC by pathological diagnosis and had not undergone any previous treatment. Patients with other malignancies were excluded. The study protocol was approved by the Ethics Committee of the Affiliated Cancer Hospital of Guangxi Medical University. All patients and their guardians were informed and signed the informed consent form.

### Cell culture

HOK cells were cultured in Oral Keratinocyte Medium (OKM, ScienCell Research Laboratories, Carlsbad, CA, USA). NP69 cells were cultured in keratinocyte serum-free medium (Defined Keratinocyte-SFM, Gibco, Carlsbad, CA). CNE-2, C666-1 and SCC-4 cells were cultured in DMEM medium (Gibco, Carlsbad, CA) supplemented with 10% fetal bovine serum (FBS), 100 U/ml penicillin and 100ug/ml streptomycin at 37°C in a humidified atmosphere of 5% CO_2_.

### Verification of the hub RNAs using RT-qPCR

The hub RNAs PART1, MIR4435-2 HG and LINC01980 were selected from the ceRNA network and subjected to RT-qPCR analysis to verify the reliability of the bioinformatics findings. The intracellular expression levels of PART1, MIR4435-2 HG and LINC01980 and their expression levels in serum were determined using RT-qPCR. Total cellular RNA was extracted using TrizolTM reagent (Takara, Dalian, Japan). Total serum RNA was extracted using miRcute Serum/Plasma miRNA isolation kit (DP160526, Tiangen Biotech, China). Total RNA was reversely transcribed into cDNA following the manufacturer’s instructions of MightyScript First Strand cDNA Synthesis Master Mix reagent kit (B639251, Sango Biotech, China). The 2X SG Fast qPCR Master Mix kit was employed, and spectrophotometer was used to determine the Ct value for each sample. GAPDH expression was measured and served as endogenous control. The measurement was repeated 3 times (n = 3) for each sample, and the data was analyzed using the 2^−ΔΔct^ method. The following primer sequences were used: (5ʹ3ʹ): MIR4435-2 HG-F: AGGAGGCGGAGCATGGAACTC, MIR4435-2HG-R: CAGGGGAAGCAAGTCTCACACATC, LINC01980-F: CATTGTAGGTGGGTGGGTGACTTC, LINC01980-R: CACTAACACAGGCTGAGCAGACTC, PART1-F: CCAGAGCCAGCCAATCACTTAGC, PART1-R: TGTTGTTCCAGTGCAGCCCTTTC.

### Statistical analysis

R software was used for all data analysis and processing. Independent t-test and ANOVA were used for continuous variables. Pearson’s chi-square test was used for categorical variables. Kaplan-Meier method was used for survival analysis. P < 0.05 was considered statistically significant. Benjamini–Hochberg procedure for multiple testing and false discovery rate (FDR) were used to correct the p-values for the selection of differentially expressed mRNAs, miRNAs and lncRNAs.

### Results

In this study, a lncRNA-miRNA-mRNA ceRNA network consisting of 90 DEGs, 67 DELs and 7 DEMs was constructed based on RNA-seq data of HNSCC from the TCGA database. WCGNA was then conducted to identify the co-expression modules of HNSCC-related lncRNAs and their associations with clinical traits. After intersecting the survival-related co-expression module with the constructed ceRNA network, the top five most significant survival-related lncRNAs (MIR4435-2HG, CASC9, LINC01980, STARD4-AS1, MIR99AHG) were yielded. Besides, lncRNA (PART1) showed the most significant correlation with HNSCC tumor. The expressions of PART1, MIR4435-2HG and LINC01980 were validated in nasopharyngeal carcinoma cell lines, oral squamous cell carcinoma cell lines and the sera of HNSCC patients using RT-qPCR assays.

### Differentially expressed mRNA, miRNA and lncRNA in HNSCC

**Figure 1.**
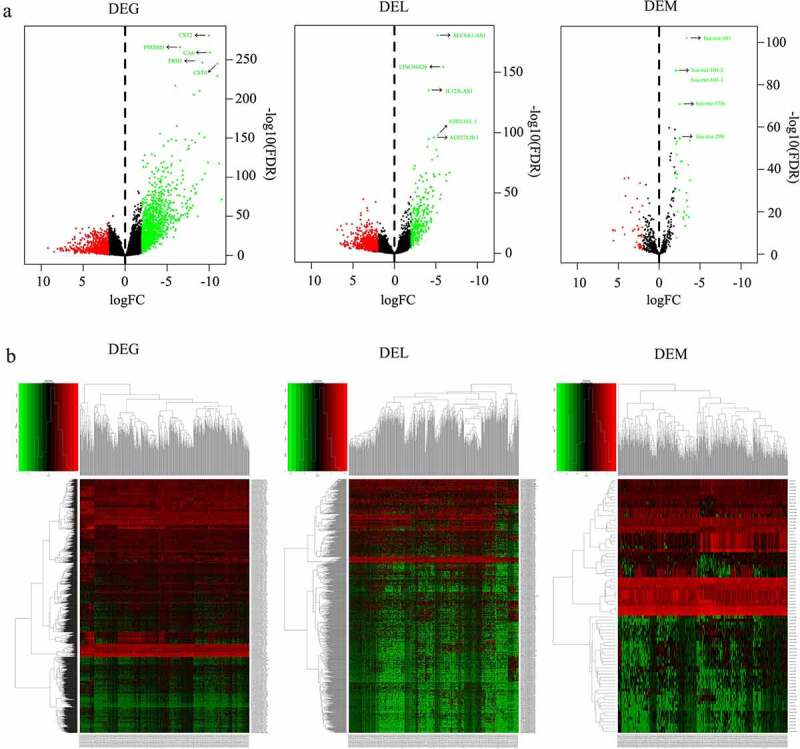
Differentially expressed RNAs in HNSCC. (a) Volcano plots of differentially expressed mRNAs (DEGs), lncRNAs (DELs) and miRNAs (DEMs) based on TCGA database. X axis represented the mean expression differences of mRNAs, lncRNAs and miRNAs between HNSCC tissues and paracancerous normal tissues, and Y axis represented log transformed false discovery rate (FDR). Red dots represented upregulated RNAs, green represented downregulated RNAs, and black represented no significant difference. The top 5 most significantly differentially expressed DEGs, DELs and DEMs were annotated in the corresponding plots. (b) Clustering heatmaps based on the expressions of DEGs, DELs and DEMs between HNSCC tissues and paracancerous normal tissues. The right vertical axis represented differentially expressed RNAs, the left vertical axis represented the sample clusters, and the bottom horizontal axis showed the TCGA sample IDs. The color scales on the upper left showed the expression values. Red color represented upregulated RNAs and green represented downregulated RNAs. DEG, differentially expressed mRNA. DEL, differentially expressed lncRNA. DEM, differentially expressed miRNA. HNSCC, head and neck squamous cell carcinoma

**Figure 2.**
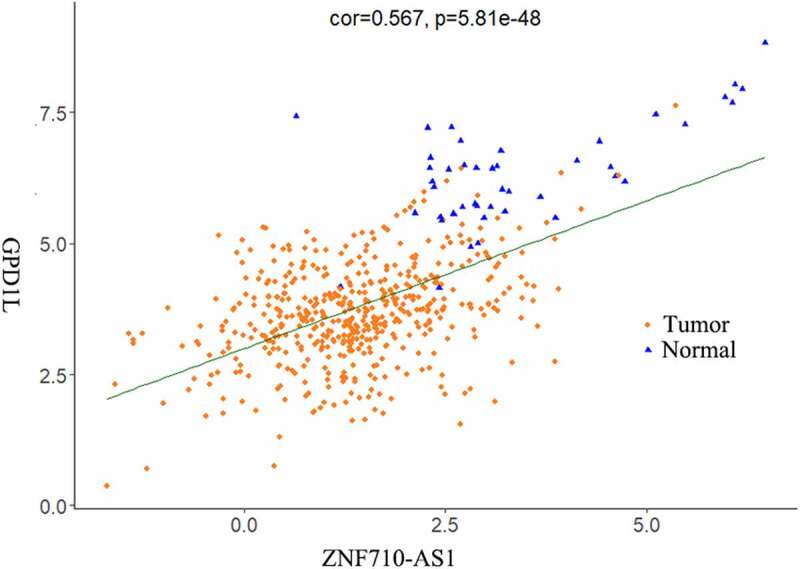
Scatter plot of the expression of lncRNA ZNF710-AS1 and mRNA GDP1L in HNSCC and normal tissues. The lncRNA-mRNA exhibited similar expression pattern in HNSCC tissues and non-HNSCC normal tissues and showed the strongest correlation in the Pearson correlation analysis of all the 1998 DEGs, 80 DEMs, 1019 DELs

According to the |log2 FC|>1 and FDR<0.01 cutoff criteria, a total of 1998 DEGs (1170 down-regulated, 828 up-regulated), 80 DEMs (33 down-regulated, 47 up-regulated), and 1019 DELs (296 up-regulated, 723 down-regulated) were identified from 519 HNSCC tissue samples and 44 non-HNSCC normal tissues from the TCGA database. The distributions of DEGs, DEMs and DELs were visualized using volcano plots (Figure 1(a)). Additionally, the top 5 most statistically significant DEGs, DEMs and DELs were summarized in Table 1 and indicated with arrows in Figure 1(a). Notably, all these most significantly differentially expressed genes were downregulated in HNSCC tissues compared to non-HNSCC normal tissues (Table 1). Furthermore, the expression levels of DEGs, DEMs and DELs between HNSCC tissues and non-HNSCC normal tissues were visualized using heatmap (Figure 1(b)). The lncRNA-mRNA pair with the strongest correlation (R = 0.567, P = 5.81e-48) was identified using Pearson correlation analysis and the relationship between their expression levels in HNSCC and normal tissues was plotted in Figure 2.

**Table 1.** Top 5 most significantly differentially expressed lncRNA, mRNA and miRNA

Symbol	Type	logFC	FDR	P-value	Expression pattern
SLC8A1-AS1	lncRNA	−5.177	3.39E-181	4.54E-185	Down
LINC01829	lncRNA	−5.894	4.63E-155	1.24E-158	Down
IL12A-AS1	lncRNA	−4.144	1.08E-135	4.36E-139	Down
AC005165.1	lncRNA	−5.235	6.18E-99	3.31E-102	Down
AC027130.1	lncRNA	−4.757	1.19E-96	7.98E-100	Down
CST2	mRNA	−9.984	1.14E-281	6.32E-286	Down
PM20D1	mRNA	−6.545	5.81E-267	6.44E-271	Down
CA6	mRNA	−10.096	5.11E-260	8.50E-264	Down
PRH1	mRNA	−9.217	3.30E-247	7.32E-251	Down
CST4	mRNA	−11.020	1.12E-245	3.10E-249	Down
hsa-mir-381	miRNA	−3.387	3.51E-103	5.42E-106	Down
hsa-mir-101-2	miRNA	−2.079	6.05E-88	1.87E-90	Down
hsa-mir-101-1	miRNA	−2.077	1.34E-87	6.20E-90	Down
hsa-mir-378 c	miRNA	−2.538	7.49E-72	4.63E-74	Down
hsa-mir-299	miRNA	−2.520	7.11E-56	7.69E-58	Down

FDR, false discovery rate. Log(FC), log fold change.

### Functional analysis for the DEGs

**Figure 3.**
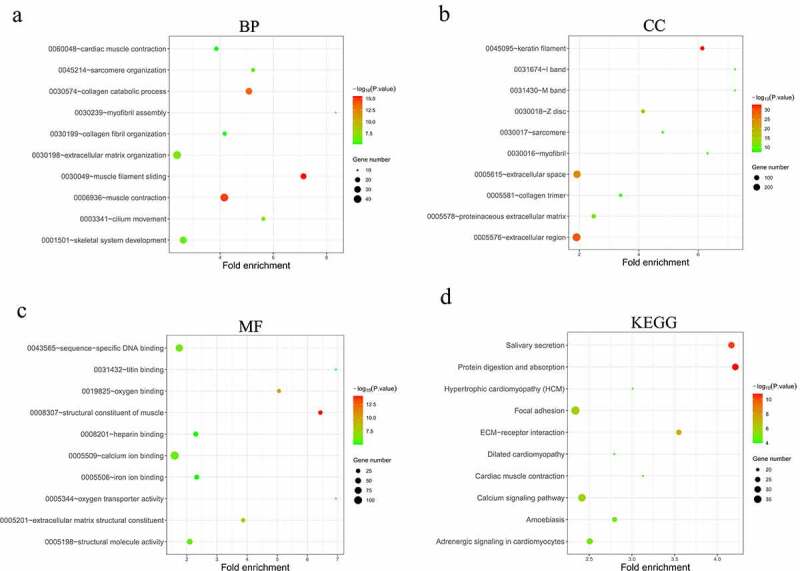
Functional analysis of the 1998 significantly differentially expressed mRNAs in HNSCC. (a-c) Top 10 most significantly enriched BP (a), CC (b), MF (c) terms of the differentially expressed mRNAs analyzed by GO enrichment analysis (*p* < 0.01). (d) Top 10 most significantly enriched KEGG pathways of the differentially expressed mRNAs (*p* < 0.01). BP, biological processes. CC, cellular components. MF, molecular functions. GO, gene ontology. KEGG, Kyoto Encyclopedia of Genes and Genomes

To obtain a global picture of the biological functions of the identified DEGs in HNSCC, GO and KEGG enrichment analyses were performed for the above identified 1998 DEGs. The top 10 most significant (p < 0.01) GO terms (BPs, CCs and MFs) and the top 10 most significantly (p < 0.01) enriched KEGG pathways were displayed in Figure 3. It is noteworthy that the analyzed DEGs were enriched in extracellular matrix organization and collagen catabolic process, which are biological processes potentially associated with cancer cell invasion and migration. Enriched CC terms indicated that the DEGs were mainly concentrated in muscle components and in extracellular regions. MF terms such as sequence-specific DNA binding and calcium ion binding indicated the potential functions of the DEGs in gene regulation and signal transduction. KEGG pathway analysis further detected several well-known cancer-associated pathways including focal adhesion, ECM-receptor interaction and calcium signaling pathways.

### Construction of lncRNA-miRNA-mRNA ceRNA networks

**Figure 4.**
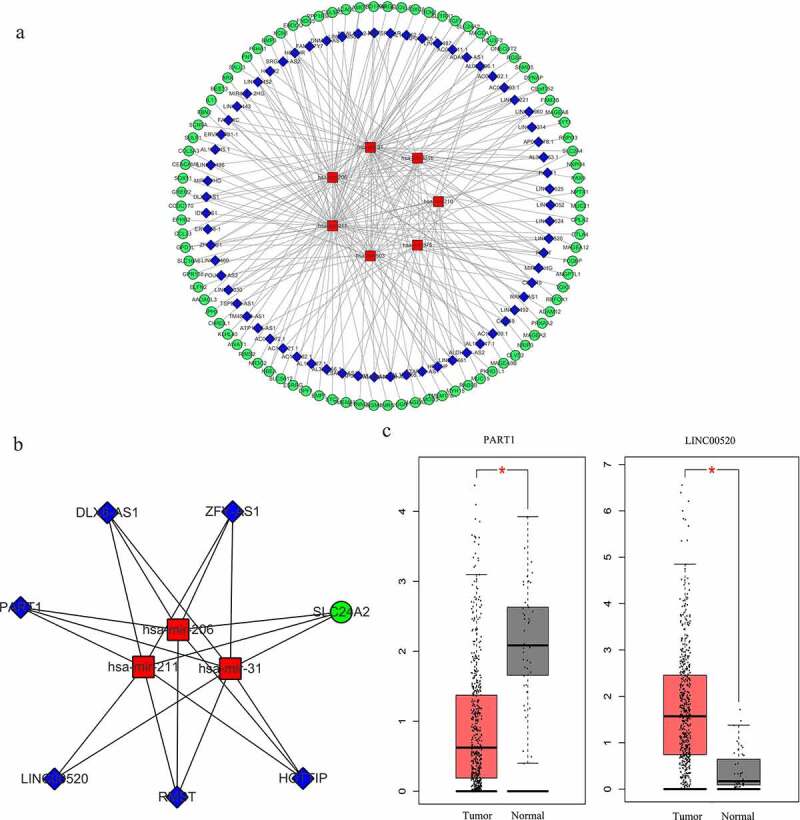
Construction of the lncRNA-miRNA-mRNA interactive ceRNA networks. (a) ceRNA network in HNSCC. A total of 164 genes including 90 DEGs, 67 DELs and 7 DEMs were used to construct the ceRNA network. (b) A ceRNA subnetwork was constructed with the top 10 hub genes showing the highest MCC scores. Green, blue and red nodes represented DEGs, DELs and DEMs, respectively. An edge in the networks represented the interactive relationship between two genes. MCC, Maximal Clique Centrality. (c) The expression of lncRNA PART1 and LINC00520 in HNSCC and normal tissues (*p* < 0.01)

**Figure 5.**
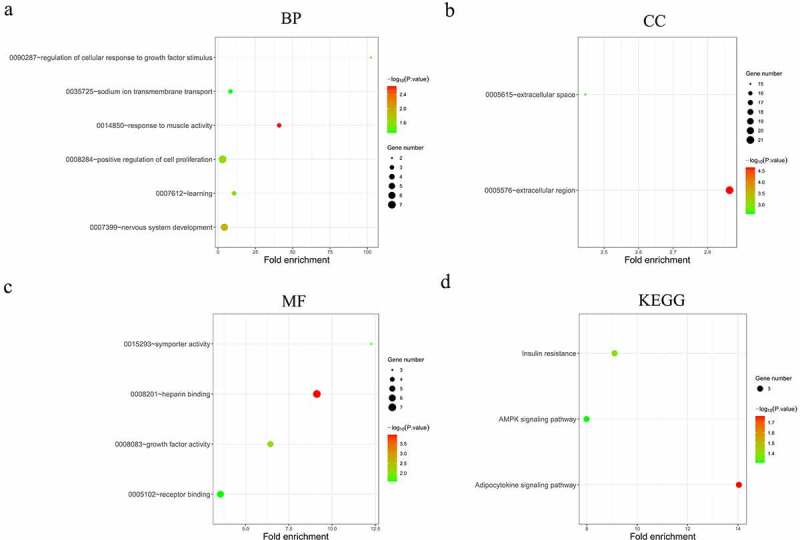
Functional analysis of the 90 significantly differentially expressed mRNAs from the constructed ceRNA network. (a-c) Top 10 most significantly enriched BP (a), CC (b), MF (c) terms of the differentially expressed mRNAs analyzed by GO (*p* < 0.01). (d) Top 10 most significantly enriched KEGG pathways of the differentially expressed mRNAs (*p* < 0.01). BP, biological processes. CC, cellular components. MF, molecular functions. GO, gene ontology. KEGG, Kyoto Encyclopedia of Genes and Genomes

From the previously obtained 80 DEMs and 1019 DELs, 136 pairs of interacting lncRNAs (including 67 HNSCC-specific DELs) and miRNAs (including 7 HNSCC-specific DEMs) were identified using miRcode database and StarBase database. MiRDB and TargetScan databases were used to predict the target genes of the 7 HNSCC-specific DEMs, and the overlap between these genes and the previously identified 1998 DEGs was analyzed, yielding 90 overlapping DEGs in total. Then, a lncRNA-miRNA-mRNA interactive ceRNA network consisting of the overlapping 90 DEGs, 67 HNSCC-specific DELs and 7 HNSCC-specific DEMs was constructed and visualized using Cytoscape (Figure 4(a)). GO and KEGG enrichment analyses were performed to investigate the biological functions of the 90 DEGs in the ceRNA network. As shown in Figure 5(a), the construction of ceRNA network has filtered out a large proportion of genes not related to cancer, as more cancer-related BPs and MFs were detected by the GO enrichment analysis. These cancer-related processes and functions included the regulation of cellular response to growth factor stimulus, positive regulation of cell proliferation, receptor binding, heparin binding and growth factor activity (Figure 5(a,c)). Extracellular region was still indicated as the predominant location where the DEGs exert their biological functions (Figure 5(b)). Moreover, KEGG enrichment results demonstrated that the DEGs were significantly enriched in the AMPK signaling pathway (Figure 5(d)), which is a well-established regulatory pathway of cellular energy homeostasis that is frequently dysregulated in tumor cells. Subsequently, the top 10 hub RNAs with the highest Maximal Clique Centrality (MCC) scores were identified using the CytoHubba plugin of Cytoscape (Table 2), which consisted of 3 DEMs, 5 DELs and 1 DEG. These hub RNAs were then used for the construction of a ceRNA subnetwork, as shown in Figure 4(b). In this subnetwork, lncRNA PART1 and LINC00520 exhibited the most significant differential expression in HNSCC tissues compared to non-HNSCC normal tissues. The expression analysis based on GEPIA database showed that PART1 was significantly downregulated and LINC00520 was significantly upregulated in HNSCC tissues (Figure 4(c)), which were consistent with the results obtained in this study (Table 2).

**Table 2.** The top 10 hub RNAs in the ceRNA network ranked by MCC scores

Rank	Name	RNA type	MCC score	Expression pattern
1	hsa-mir-211	miRNA	102.6666667	Down
2	hsa-mir-31	miRNA	92	Up
3	hsa-mir-206	miRNA	84.01666667	Down
4	RMST	lncRNA	81.16,666,667	Down
5	DLX6-AS1	lncRNA	79.75	Up
6	HOTTIP	lncRNA	79.25	Up
7	ZFY-AS1	lncRNA	79.25	Down
8	PART1	lncRNA	79.25	Down
9	SLC24A2	mRNA	77.08333333	Up
10	LINC00520	lncRNA	75.75	Up

MCC, Maximal Clique Centrality.

### Weighted gene co-expression network analysis of lncRNAs

**Figure 6.**
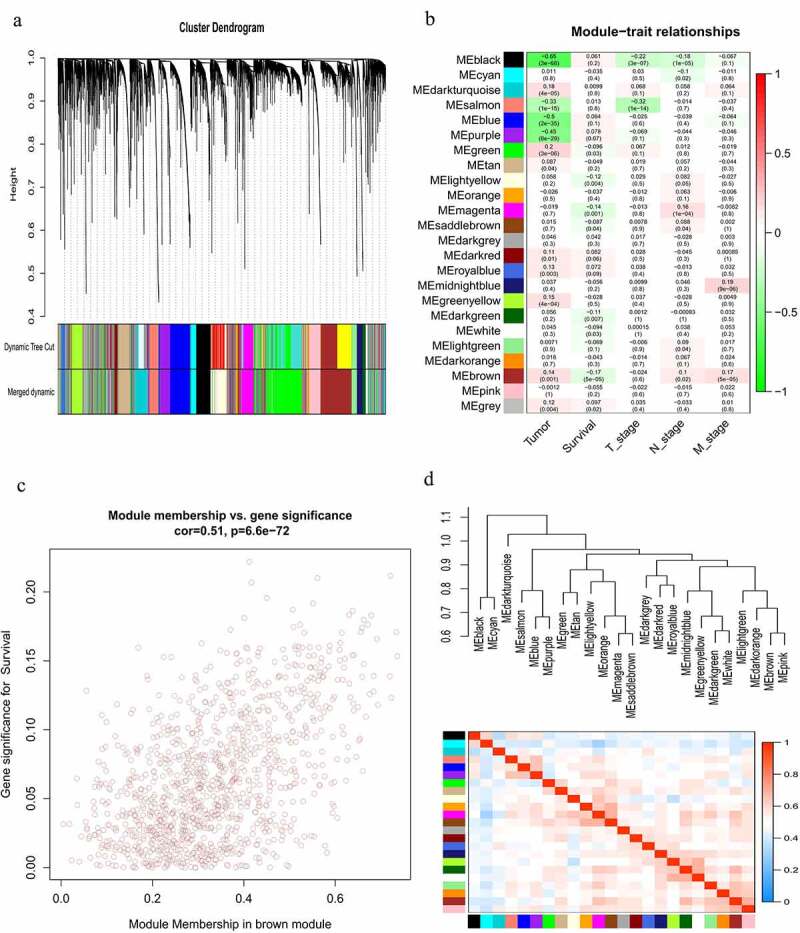
Construction of a lncRNA co-expression network using WGCNA. (a) Cluster dendrogram of the co-expression network for the top 60% variance lncRNAs based on topological overlap. (b) Analysis of the correlations of co-expression modules with clinical traits. The background color of each cell represented the strength of correlation between the gene module and the clinical traits. Each cell contains two values: the value above is the Pearson’s correlation coefficient and the value in parentheses below is the P-value. (c) Scatter diagram for the correlation between module membership and gene significance for survival in the brown module. (d) Heatmap plot of the adjacencies in the gene co-expression network

To identify the gene modules that are significantly correlated with overall survival (OS) of HNSCC patients, a co-expression network of the top 60% variance lncRNAs was constructed using WGCNA algorithm. Hybrid hierarchical clustering based on the topological overlaps was generated using the Dynamic Tree Cut method (Figure 6(a)). By setting the power (β) = 3 as soft threshold and comparing the Pearson’s correlation coefficients (PCC), 24 co-expression modules of lncRNAs associated with clinical traits were identified (Figure 6(b)). Among them, the gray module consisted of genes that were not co-expressed and was therefore not included in further analysis. Of note, the brown module showed the most significant correlation with the survival of HNSCC patients (R = 0.17, P = 5e−05). The correlation between module membership (MM) in the brown module and gene significance (GS) for survival was shown in Figure 6(c). To further compare the similarity of co-expression levels, the modules were clustered according to the correlation with module eigengenes (MEs) (Figure 6(d)). Finally, the lncRNAs in the brown module were intersected with the 67 differentially expressed lncRNAs in the constructed ceRNA network to ascertain the prognostic value of lncRNAs in the constructed ceRNA network, and to filter out all survival-related lncRNAs that were also significantly differentially expressed in HNSCC tissues. Notably, 5 survival-related lncRNAs from the brown module were also found in the ceRNA network (Table 3), which were subsequently subjected to Kaplan-Meier survival analysis. Moreover, lncRNA PART1 from the purple module exhibited the most significant correlation with HNSCC (p.GS.tumor = 3.69E-33) (Table 3) across all modules in the co-expression network and was also present in the previously constructed ceRNA network and subnetwork.

**Table 3.** Differentially expressed lncRNAs that were highly correlated with HNSCC and patient survival

Probes	moduleColor	GS.Tumor	p.GS.Tumor	GS.Survival	p.GS.Survival
PART1	purple	−0.482	3.69E-33	0.054	0.206
MIR4435-2HG	brown	0.216	3.41E-07	−0.212	5.59E-07
CASC9	brown	0.182	1.89E-05	−0.159	0.0001
LINC01980	brown	0.225	1.12E-07	−0.117	0.006
STARD4-AS1	brown	0.183	1.75E-05	0.161	0.0002
MIR99AHG	brown	0.083	0.043	0.172	4.83E-05

GS, gene significance.

### Verification of the prognostic values of lncRNAs

**Figure 7.**
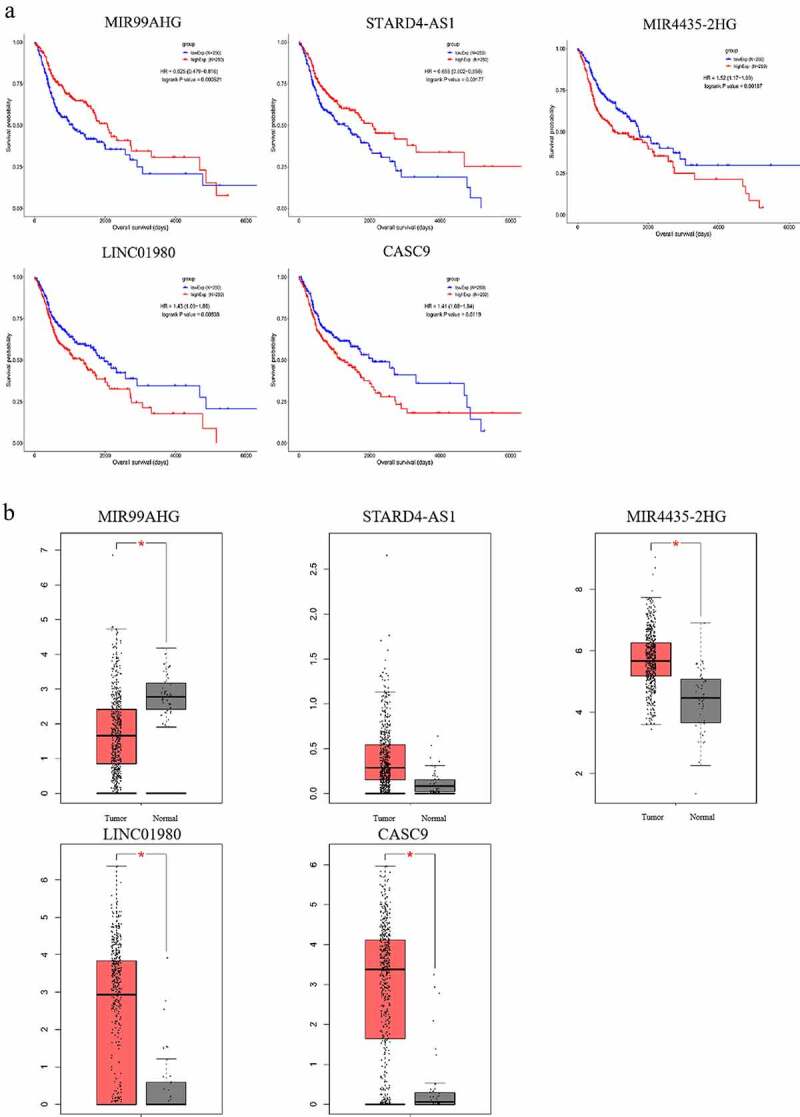
Kaplan-Meier survival analysis and the expression profiles of the 5 survival-associated lncRNAs. (a) Kaplan-Meier survival curves of the 5 survival-associated lncRNAs. X axis represented the overall survival time in months. Y axis represented the survival probability. The logrank test was used to compare the survival time between the low- and high-expression groups (*p* < 0.05). (b) Expression levels of survival-related lncRNAs in HNSCC tissues and non-HNSCC normal tissues (*p* < 0.01)

**Figure 8.**
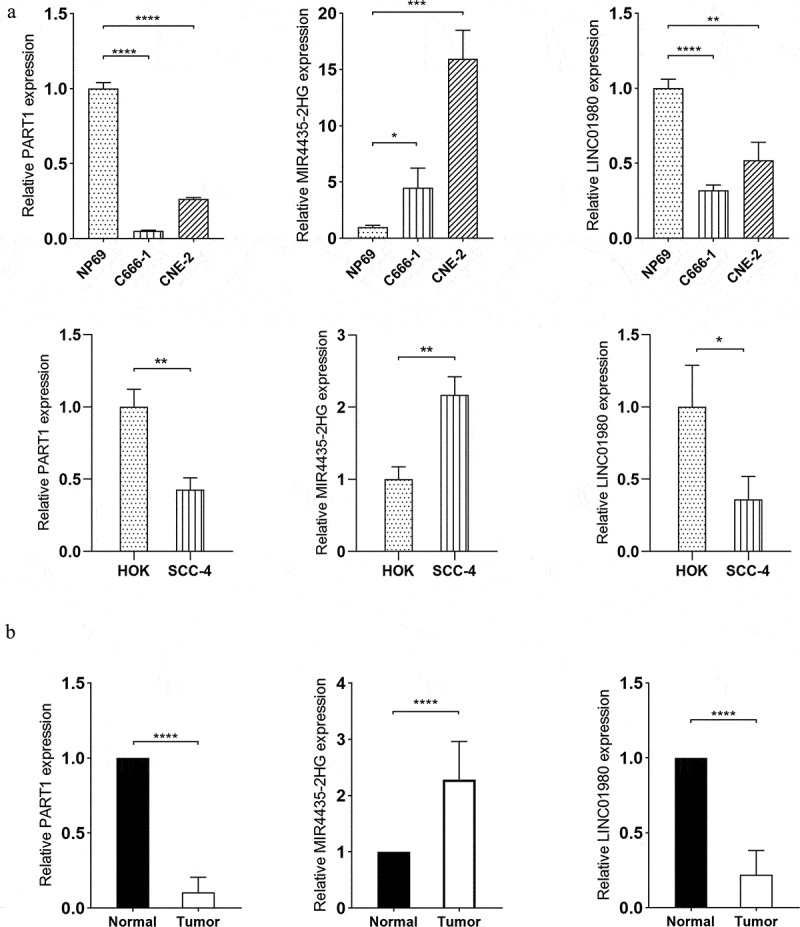
Verification of expression patterns of lncRNA PART1, MIR4435-2H and LINC01980 in HNSCC. (a) Total RNA extracted from NP69 (immortalized nasopharyngeal epithelial cell line), C666-1 (nasopharyngeal carcinoma cell line), CNE-2 (nasopharyngeal carcinoma cell line), HOK (human oral keratinocytes) and SCC-2 (OSCC cell line) was subjected to RT-qPCR assays to determine the expression levels of PART1, MIR4435-2H and LINC01980. (b) Total RNA extracted from the serum of normal individuals and HNSCC patients was subjected to RT-qPCR assays to determine the expression levels of PART1, MIR4435-2H and LINC01980. *p < 0.05

To evaluate the prognostic value of the constructed ceRNA network, the 5 lncRNAs (MIR4435-2HG, CASC9, LINC01980, STARD4-AS1, MIR99AHG) with the strongest correlation with the survival of HNSCC patients (Table 3) were subjected to Kaplan-Meier survival analysis. The survival curves of the 5 lncRNAs were shown in Figure 7. Among the analyzed genes, patients overexpressing MIR4435-2HG, LINC01980, and CASC9 showed a significantly decreased overall survival (p < 0.05), whereas patients carrying overexpression of MIR99AHG and STARD4-AS1 exhibited a superior prognosis (p < 0.05). Furthermore, the expression of these 5 lncRNAs in HNSCC tissues and non-HNSCC normal tissues were validated using GEPIA database (Figure 8). While 4 out of 5 lncRNAs showed significant differential expression, the expression of STARD4-AS1 failed to reach statistical significance between tumor and normal tissues, probably due to changing the databases.

### Validation of gene expressions of diagnostic and prognostic DELs

To verify the reliability of the bioinformatics findings, two survival-related DELs (MIR4435-2H and LINC01980) and the HNSCC-related DEL (PART1) were analyzed by RT-qPCR assays to validate their expression patterns. Consistent with our *in silico* findings, the expression of MIR4435-2H was upregulated and the expressions of PART1 and LINC0198 were downregulated in nasopharyngeal carcinoma cells (C666-1 and CNE-2) and OSCC cells (SCC-4) compared to their respective controls (HP69 and HOK) (Figure 8(a)). Similar expression patterns of the three DELs were further confirmed in the serum of HNSCC patients compared to normal individuals (Figure 8(b)). Collectively, the RT-qPCR results were in line with the above-described bioinformatics findings, thus confirming the validity and reliability of the bioinformatics analyses performed.

## Discussion

Excavating novel diagnostic and prognostic biomarkers is a pressing need for the ambition of earlier detection of preneoplastic tumors and personalized treatment designs for HNSCC patients. Recent years, lncRNAs have drawn increasing attention and are considered as highly informative biomarkers in the oncology setting. According to the ceRNA hypothesis first founded by Salmena et al., lncRNAs can fine-tune numerous biological processes via modulating miRNA activities and the miRNA-regulated mRNA expression [14]. Mounting evidence has demonstrated that lncRNAs are not only inextricably associated with tumorigenic processes such as tumor cell proliferation, invasion, migration and metastasis [7,36,37], but are also detectable in body fluids [38,39], making them a promising biomarker in reflecting the pathological status of the disease. Therefore, in this study, a series of bioinformatics approaches, including the construction and analysis of ceRNA networks, GO and KEGG enrichment analyses, WGCNA and Kaplan-Meier survival analyses, were integrated to systematically analyze the RNA-seq data of HNSCC samples from TCGA database for the sake of identifying novel cancer-related lncRNAs with diagnostic and prognostic potentials.

A total of 1998 DEGs, 80 DEMs and 1019 DELs were identified in HNSCC tissues. The DEGs were significantly enriched in several cancer-associated processes and pathways previously reported to be essential for the cancer invasion-metastasis cascade, such as collagen catabolic process [40], extracellular organization [41], focal adhesion and ECM-receptor interaction [40]. Further functional analyses of the 90 DEGs from the constructed ceRNA network revealed that the genes were crucially involved in sustaining the proliferative signaling of cancer cells. Moreover, KEGG enrichment results showed that the 90 DEGs were significantly enriched in the AMPK signaling pathway, which is a well-established cancer-related pathway essential for the survival of cancer cells under metabolic stress [42,43]. Apart from this, due to the strong correlation of these DEGs with other nodes within the ceRNA network, it is well-reasoned to speculate that miRNAs and lncRNAs in this network may also play a role in similar biological processes and pathways.

LncRNA PART1 (prostate androgen-regulated transcript 1) was originally considered as a tumor suppressor gene for prostate cancer due to its location on the chromosome 5q12.1^45^. This chromosome region has been demonstrated to be associated with tumor-suppressive activities and was found to be deleted in prostate cancer cells and 39% of prostate cancer metastases analyzed [44]. However, functional studies of some other solid malignancies have reported opposite findings suggestive for an oncogenic role of PART1. For example, recent *in vitro* observations indicated that silencing of PART1 substantially sensitized the response of TE1 and KYSE-450 esophageal squamous cell carcinoma (ESCC) cells to gefitinib, while upregulation of PART1 led to sequestering of miR-129 and the resultant upregulation of Bcl-2, thereby conferring ESCC cells resistance to gefitinib [45]. Investigations in non-small cell lung cancer and colorectal cancer also showed that overexpression of PART1 markedly promoted tumor cell proliferation and epithelial–mesenchymal transition *in vitro* and accelerated tumor growth in mouse xenograft models *in vivo* [46,47]. By contrast, in the context of HNSCC, bioinformatics findings in tongue squamous cell carcinoma (TSCC) supported the tumor suppressive role of PART1, demonstrating that PART1 was downregulated in TSCC tissues and that high expression levels of PART1 were associated with a prolonged overall survival of TSCC patients [48,49]. Consistent with these results, in this study, PART1 was found to be a downregulated DEL in HNSCC tissues, and its differential expression exhibited the highest statistical significance among all genes in the ceRNA subnetwork. RT-qPCR data of HNSCC cell lines and the sera of HNSCC patients further confirmed the downregulated expression pattern of PART1 in HNSCC. Furthermore, miRNA hsa-mir-211, hsa-mir-206 and hsa-mir-31 showed a significant correlation with PART1 according to the ceRNA subnetwork. This provided novel clues about possible initial molecular events through which PART1 exerts its functions, and these miRNAs may therefore serve as a starting point to dissect the mechanism of action of PART1 in HNSCC cells. Most importantly, PART1 showed the strongest correlation with HNSCC tumor across all co-expression modules constructed by WCGNA, suggesting its potential as a diagnostic biomarker to detect preneoplastic HNSCC earlier.

To further explore prognosis-related lncRNAs for HNSCC, intersection was taken between the survival-related brown co-expression module and the constructed ceRNA network, yielding a set of 5 survival-related DELs: MIR4435-2HG, CASC9, LINC01980, STARD4-AS1 and MIR99AHG. Kaplan-Meier survival analysis indicated that high expressions of MIR4435-2HG, LINC01980, and CASC9 and low expressions of STARD4-AS1 and MIR99AHG were correlated with a significantly reduced overall survival time of HNSCC patients. Therefore, these five lncRNAs can be considered to have prognostic values for the survival of HNSCC patients, and additional clinical evaluations are needed to further confirm their predictive power. In addition, RT-qPCR analysis of HNSCC cells and patient serum verified the overexpression of MIR4435-2HG or LINC01980.

A variety of cancers aberrantly express MIR4435-2HG; its overexpression has been observed in renal [50], gastric [51], colorectal [52], brain [53], ovarian [54], lung [55] and liver tumors [56] and has been functionally linked to a highly proliferative and invasive phenotype. Elevated MIR4435-2HG expression was also detected in TSCC cell lines CAL27 and SCC25, whereas knockdown of MIR4435-2HG attenuated its inhibition on miR-383-5p, leading to downregulation of RNA‑binding motif protein 3 (RBM3) and an impaired cell proliferation and invasion [57]. The results in this study further corroborated that MIR4435-2HG was highly expressed in HNSCC tissues, cell lines and patient sera and is clinically associated with an inferior prognosis. Intriguingly, recent transcriptomics studies based on TCGA database showed that dysregulated MIR4435-2HG was closely associated with tumor cell glycolysis in hepatocellular carcinoma, mesothelioma, glioma, bladder carcinoma, pancreatic adenocarcinoma and uveal melanoma [56]. These new clues suggest that MIR4435-2HG could also act as an important regulator in deregulating cellular energetics, which is another hallmark of cancer [58]. However, to date, the function of MIR4435-2HG in glycolysis remained unexplored in HNSCC, and further functional studies are still urgently awaited to unravel the mechanistic basis underlying this *in silico* observation.

At present, there is very limited evidence for the implication of lncRNA LINC01980 in cancer, and its role in HNSCC has not been documented to date. Previous investigations in ESCC have detected a significantly elevated expression of LINC01980 in ESCC cell lines and patient-derived tissue specimens [59,60]. Observational follow-up data further demonstrated that LINC01980 overexpression was correlated with a decreased overall survival of ESCC patients [59,60]. Mechanistically, forced (over)expression of LINC01980 in ESCC cell lines has been shown to promote Myosin-Va (MYO5A) expression by inhibiting miR-190a-5p, resulting in enhanced tumor cell proliferation and EMT [59]. Upregulated expression and tumorigenic potential of LINC01980 has also been observed in hepatocellular carcinoma cell lines Huh7 and Hep3B [61]. In line with these findings, in this study, LINC01980 overexpression was detected in HNSCC tissues, cell lines and patient sera by bioinformatics approaches and RT-qPCR assays, which was also confirmed to be correlated with a significantly reduced overall survival time. The mechanistic rationale of LINC01980 in HNSCC still warrants further investigation, and the DEMs and DEGs in the constructed ceRNA network in this study may serve as an attractive starting point for these future functional studies.

In summary, this study successfully constructed a lncRNA-miRNA-mRNA interactive ceRNA network for HNSCC and identified a set of lncRNAs with diagnostic and prognostic values for HNSCC patients. These identified lncRNAs may therefore hold the promise of further refining the accuracy and explanatory power of the existing diagnostic and prognostic tools. To the best of our knowledge, this is the first study to excavate novel diagnosis- or prognosis-related lncRNAs in HNSCC through constructing ceRNA networks combined with WGCNA. However, several limitations to the present study still need to be noted. First, the findings in this study were based on the TCGA database with pure in silico methods. Although some findings have been validated by the GEPIA database and RT-qPCR analysis, additional confirmations using experimental methods are still needed. Second, the group of patients enrolled in this study is relatively small, and the cell lines used for validation did not cover all possible anatomical origins of HNSCC, suggesting that additional studies in larger patient cohorts and in more different HNSCC cell lines are needed to yield more conclusive data on the accuracy and robustness of the identified biomarker candidates. Nonetheless, translating these lncRNAs into clinical applications or developing novel therapeutic strategies targeting these lncRNAs both require future functional studies to elucidate the complete picture of their molecular mechanisms. The RNA interactions in the constructed ceRNA networks in this study may provide valuable hints to initiate these studies.

## Conclusions

In summary, the *in silico* findings of our integrated bioinformatics analysis combining ceRNA network construction with WGCNA identified five differentially expressed lncRNAs (MIR4435-2HG, CASC9, LINC01980, STARD4-AS1 and MIR99AHG) with pronounced correlation with OS of HNSCC patients and one lncRNA (PART1) with a superior performance in discriminating HNSCC tissues from non-HNSCC normal tissues. The credibility of these computational findings was further confirmed by a series of RT-qPCR analyses, which detected similar expression patterns of PART1, MIR4435-2HG and LINC01980 in HNSCC cell lines and patient sera.

## Supplementary Material

Supplemental Material

## Data Availability

The data that support the findings of this study are available from the corresponding author upon reasonable request.

## Research highlights

- The lncRNA MIR4435-2H was upregulated and PART1 and LINC0198 were downregulated in HNSCC.
- PART1 showed diagnostic potential in discriminating HNSCC from normal tissues.
- LncRNAs MIR4435-2HG and LINC01980 were significantly correlated with OS of HNSCC patients.
